# Intravascular Lithotripsy for Calcium Modification in Chronic Total Occlusion Percutaneous Coronary Intervention

**DOI:** 10.1155/2021/9958035

**Published:** 2021-06-21

**Authors:** Anja Øksnes, Claudia Cosgrove, Simon Walsh, Kjetil Halvorsen Løland, Jack Laffan, Sinjini Biswas, Aadil Shaukat, Colm Hanratty, Julian Strange, James C. S. Spratt, Margaret McEntegart

**Affiliations:** ^1^Haukeland University Hospital, Bergen, Norway; ^2^St George's University Hospital, London, UK; ^3^Belfast Health and Social Care Trust, Belfast, UK; ^4^Bristol University Hospital, Bristol, UK; ^5^Golden Jubilee National Hospital, Glasgow, UK

## Abstract

Intravascular lithotripsy (IVL) has been shown to be safe and effective for calcium modification in nonocclusive coronary artery disease (CAD), but there are only case reports of its use in calcified chronic total occlusions (CTO). We report data from an international multicenter registry of IVL use during CTO percutaneous coronary intervention (PCI) and provide provisional data regarding its efficacy and safety. During the study period, IVL was used in 55 of 1053 (5.2%) CTO PCI procedures. IVL was used within the occluded segment after successful CTO crossing in 53 procedures and during incomplete CTO crossing in 2 cases. The mean J-CTO score was 3.1. CTO PCI technical and procedural success was achieved in 53 (96%) and 51 (93%) cases. Six patients had a procedural complication, with 3 main vessel perforations (5%). Two had covered stent implantation, one required pericardiocentesis, and one was managed conservatively. All had combination therapy with another calcium modification device. Two patients had a procedural myocardial infarction (PMI) (4%), and two others had a major adverse cardiovascular event (MACE) (4%) at a median follow-up of 13 (4–21) months. IVL can effectively facilitate calcium modification during CTO PCI. More data are required to establish the efficacy and safety of IVL and other calcium modification devices when used extraplaque or in combination during CTO PCI.

## 1. Introduction

Calcification within a chronic total occlusion (CTO) is an independent predictor of percutaneous coronary intervention (PCI) failure, prolonged procedure duration, suboptimal stent expansion, and complications and thus is a key characteristic in CTO complexity scores [1–4]. Calcium is most commonly located at the proximal cap and also frequently present within the occlusive segment and at the distal cap, particularly in postbypass native vessel occlusions where it is most prevalent [5].

**Figure 1:**
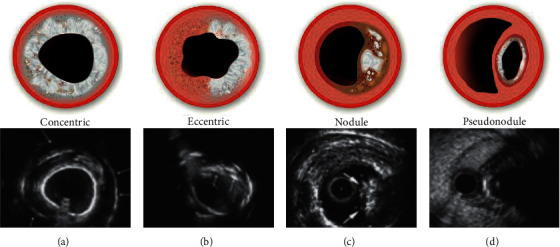
Calcium morphologies (concentric: smooth surfaced with reverberations consistent with thin calcium; eccentric: irregular surface with no reverberation consistent with thick calcium; nodule: luminal protrusion with irregular leading edge; pseudonodule: subintimal space expansion with displacement of calcified plaque and lumen).

CTOs can be crossed by luminal tracking (“intraplaque”) or by dissecting around the occlusion and through the less-resistant subintimal space (“extraplaque”). This can be done antegradely (antegrade wiring (AW) or antegrade dissection re-entry (ADR)) or retrogradely (retrograde wiring (RW) or retrograde dissection re-entry (RDR)). Calcium modification is required more often if the course is intraplaque. While the morphology of plaque calcium can be concentric, eccentric, or nodular, extraplaque tracking and displacement of the plaque and lumen can result in the formation of a calcified “pseudonodule” (Figure 1).

Calcium modification is frequently performed during CTO PCI using scoring (SB), cutting (CB), and high-pressure (OPN) balloons or rotational (RA), orbital or laser atherectomy, although there are limited published data on the efficacy and safety of these devices in this patient population [6, 7]. While the more recently available Shockwave ™ intravascular lithotripsy (IVL) balloon has been demonstrated to be safe and effective in nonocclusive CAD [8–12], there are only few case reports of its use during CTO PCI [13, 14].

We describe the initial experience of using IVL during CTO PCI and provide provisional data regarding its efficacy and safety.

## 2. Materials and Methods

We retrospectively identified patients treated with IVL within the occluded segment during CTO PCI at 5 high-volume centers in the UK and Norway.

We describe the patient demographics, clinical presentation, CTO characteristics, procedural strategy, and calcium modification techniques. The procedural angiograms and reports were systematically reviewed and adjudicated by two members of the CTO team at each site.

Procedural outcomes, complications, and inhospital major adverse cardiovascular event (MACE) rates are reported. Long-term MACE rates were determined from electronic databases. Definitions were applied according to the CTO Academic Research Consortium (CTO ARC) [15]. All patients gave consent for use of their anonymized data.

## 3. Results

During the study period, 1053 patients had CTO PCI, with IVL used for calcium modification within the occluded segment in 55 patients (5,2%). Patient demographics, clinical presentation, and CTO characteristics are described in Table 1. The CTOs were high complexity with 36% having previous coronary artery bypass grafting (CABG), 20% having a previous failed CTO PCI attempt, and with a mean J-CTO score of 3.1.

Procedural setup, final strategy, and calcium modification techniques are described in Table 2. Biradial or radial-femoral dual access was used in 80% of cases. The proportions of final successful crossing strategy were consistent with contemporary CTO registries [16, 17]. Intravascular imaging was used in almost 90% of cases.

**Figure 2:**
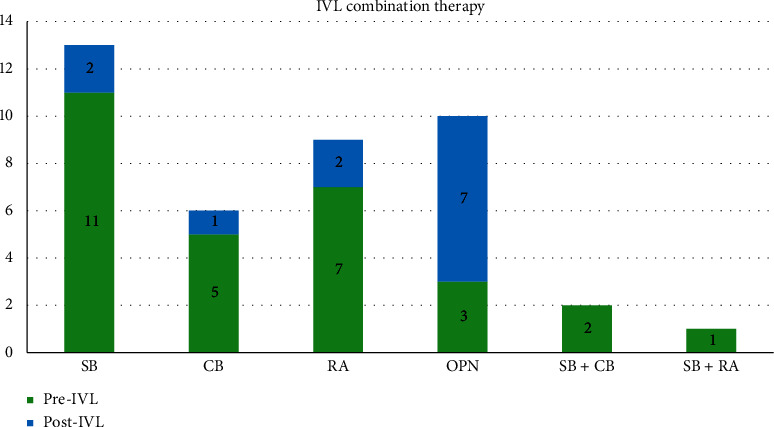
IVL use in combination therapy. SB = scoring balloon; CB = cutting balloon; OPN = high pressure balloon; RA = rotational atherectomy; IVL = intravascular lithotripsy.

**Figure 3:**
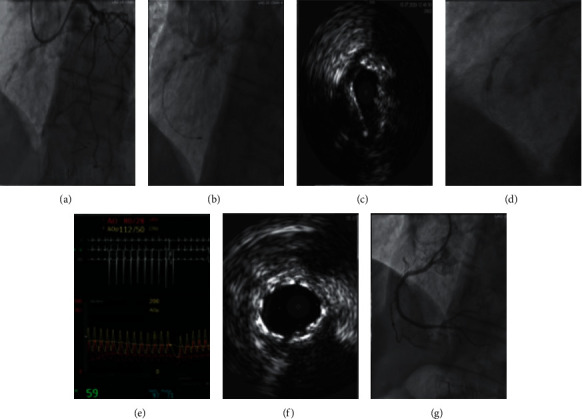
Calcium modification of mid-RCA CTO with RA and IVL. (a) Angiographic severe calcification mid-RCA; (b) RA; (c) IVUS confirmed intraplaque crossing with concentric calcium; (d) IVL with dogbone; (e) “Shocktopics” on EKG; (f) IVUS confirmed stent expansion; (g) final angiographic result.

IVL was used after successful CTO crossing and prior to stenting in 53 cases and after incomplete CTO crossing during a CTO modification procedure in 2 cases. It was used extraplaque in 35% of patients. The decision to use IVL was made by the operator after assessing plaque modification to be inadequate after initial treatment with a noncompliant (NCB), scoring (SB), cutting (CB), high-pressure balloon (OPN), or rotational atherectomy (RA). Inadequate plaque modification was determined either angiographically by observing balloon under expansion, as is routine practice, or by intravascular imaging. Lesion pretreatment was performed using an NCB in 96% of cases and an additional calcium modification device (SB, CB, OPN, or RA) in 42% of cases prior to IVL use (Figure 2). Additional calcium modification devices were used after IVL in 22% of cases (Figure 2). In total, combination therapy of IVL with another calcium modification device was required in 53% of patients.  Figure 3 illustrates a case where IVL was used following RA, demonstrating the initial underexpanded IVL balloon during delivery of the first 10 pulses of therapy and IVUS confirmation of adequate stent expansion.

CTO PCI technical and procedural success were achieved in 53 (96%) and 51 (93%) patients, respectively. Of the eleven cases that were reattempts, 5 had a CTO modification procedure at the time of the first attempt, with IVL used in 2 patients at the proximal cap and extraplaque. Both patients subsequently had successful staged CTO PCI completion (1 ADR and 1 RDR). Drug eluting balloons were used in almost 1 in 5 cases, either for instent restenosis or to treat distal disease.

There were three (5%) Ellis grade 3 main vessel perforations. One following IVUS confirmed intraplaque crossing of a tortuous right coronary artery (RCA), with RA followed by IVL, resulting in tamponade, pericardiocentesis, and covered stent implantation. The second after IVUS confirmed extraplaque crossing of a left anterior descending (LAD) artery, modified with an SB and IVL, treated with a covered stent. The third following IVUS confirmed extraplaque crossing of an RCA, with IVL (10 pulses of therapy at the perforation site) followed by CB and stenting, resulting in an atrioventricular groove hematoma managed conservatively. There was 1 femoral access site bleed requiring blood transfusion, 1 heart block requiring pacemaker implantation, and 1 septal perforation requiring coiling.

Two patients had a procedural myocardial infarction (PMI) (4%). Two other patients had target lesion revascularization (TLR) due to instent restenosis (ISR). There were no other MACE at a median follow-up of 13 (4–21) months.

## 4. Discussion

This is the first reported cohort of patients treated with IVL for calcium modification during CTO PCI. In almost all cases, IVL was used after CTO crossing, intraplaque in two-thirds, and extraplaque in one-third of patients. In over 50% of cases IVL was used in combination with another device, most commonly after incomplete calcium modification with the first device, but in 22% of cases, additional calcium modification was needed after IVL. Of the 3 main vessel perforations, all occurred where IVL was used in combination with another device, and in 2 cases extraplaque.

Following intraplaque crossing and predilatation, we would anticipate that the efficacy and safety of IVL would be the same as in nonocclusive disease. While the efficiency of its use is a potential advantage during prolonged CTO PCI procedures, atherectomy may be required for balloon uncrossable lesions or to treat extensive segments of calcific disease.

When dissection re-entry is used for CTO crossing, the technique itself results in plaque and luminal displacement with associated calcium “modification.” This can result in the formation of a “calcified pseudonodule” (Figure 1), with further modification and stenting occurring extraplaque. Inadequate modification of a calcified nodule results in eccentric stent expansion, and while debulking with atherectomy is thought to be most effective in treating intraplaque nodules, there are limited data on its efficacy and safety extraplaque [6, 7]. Indeed, without favorable wire-bias, especially when using small burrs, modification will be limited but with some associated risks. While SB, CB, OPN, and IVL are frequently used to treat nodular calcium in nonocclusive disease, their efficacy and safety has not been described. Furthermore, the efficacy and safety of all the calcium modification devices is uncertain in the treatment of extraplaque pseudonodular calcium.

IVL could potentially be used to facilitate CTO crossing, specifically for balloon-assisted subintimal entry (BASE) at a calcified proximal cap, or in connecting antegrade and retrograde spaces during RDR (14). In addition, as described in two of our cases, it may have a role during modification procedures for complex calcified CTOs [15].

The key limitation of this study is that it is a retrospective, observational cohort. In addition, it should be noted that IVL was used after CTO crossing in almost all cases and thus introduces bias to procedural success rates. A period of novel technology adoption and impact of incremental device cost will have introduced some case selection bias, increasing the proportion of cases where an additional calcium modification device was used before IVL, where with improved access and more experience, IVL could be the first choice device when initial treatment with NCB has failed. While intravascular imaging was used in 90% of the procedures, quantitative data was not available for analysis.

## 5. Conclusion

IVL can be used effectively for calcium modification during CTO PCI. More data are required to establish the efficacy and safety of IVL and other calcium modification devices during CTO PCI, especially when used extraplaque or as combination therapy.

## Figures and Tables

**Table 1:** Patient demographics and CTO characteristics.

	*N* = 55
Age (years)(mean)	70.2
Gender (F/M)	(7/48)
Clinical presentation	
Stable angina	43 (78%)
Acute coronary syndrome	11 (20%)
Asymptomatic ischemia	2 (3.6%)
Prior CABG	20 (36%)
Prior PCI	34 (61.8%)
Hypertension	36 (65.5%)
Hypercholesterolemia	39 (70.1%)
Diabetes	20 (36%)
eGFR (mean)	69
Smoking	24 (43.6%)
LVEF (%)	50%
Target vessel	
LAD	16 (29%)
RCA	33 (60%)
CX	7 (13%)
LMS	1 (2%)
>1 vessel treated	2 (3.5%)
CTO characteristics	
Calcification	55 (100%)
Ambiguous proximal cap	21 (38%)
Length >20 mm	35 (63.6%)
Tortuosity	24 (43.6%)
Ambiguous distal cap	26 (47.3%)
Instent occlusion	8 (14.5%)
Reattempt	11 (20%)
Modification procedure at 1^st^ attempt	5 (9.1%)
J-CTO score (0–5)(mean)	3.1

[CABG = coronary artery bypass grafting; PCI = percutaneous coronary intervention; eGFR = estimated glomerular filtration rate; LVEF = left ventricular ejection fraction; LAD = left anterior descending artery; RCA = right coronary artery; CX = circumflex artery; LMS = left main stem; J-CTO = Japanese-CTO score].

**Table 2:** Procedural data.

	*N* = 55
Access site	
Single radial	9 (16.4%)
Biradial	31 (56.4%)
Radial/femoral	13 (23.6%)
Bifemoral	2 (3.6%)
Dual injection angiography	46 (83.6%)
Guide catheters 5/6/7/8 French	1/8/87/5
Final strategy	
AW	32 (58.2%)
RDR	13 (23.6%)
ADR	6 (10.9%)
RW	2 (3.6%)
Unsuccessful	2 (3.6%)
IVUS	48 (87.3%)
OCT	1 (1.8%)
Pre-IVL	
NCB	53 (96.4%)
SB	11 (20%)
CB	5 (9%)
OPN	3 (5.5%)
RA	7 (12.3%)
IVL therapy	
IVL balloons (mean number/case)	1.2
Number of pulses (mean)	60
Proximal cap	51 (92.7%)
CTO body	51 (92.7%)
Extraplaque	19 (34.5%)
Guide extension delivery	28 (50.1%)
Balloon burst	7 (12.7%)
Non-CTO segment	21 (38.2%)
Bifurcation	11 (20%)
Post-IVL	
NCB	53 (96.4%)
SB	2 (3.6%)
CB	1 (1.8%)
OPN	7 (12.7%)
RA	2 (3.6%)
Number of stents (mean)	2.5
Mean stent length (mm)	83
DEB	10 (18%)

AW = antegrade wiring; RDR = retrograde dissection re-entry; ADR = antegrade dissection re-entry; RW = retrograde wiring; IVUS = intravascular ultrasound; OCT = optical coherence tomography; IVL = intravascular lithotripsy; NCB = noncompliant balloon; SB = scoring balloon; CB = cutting balloon; OPN = high-pressure balloon; RA = rotational atherectomy; DEB = drug eluting balloon.

## Data Availability

The data used to support the findings of this study are included within the article.
